# Autism spectrum disorder in females with fragile X syndrome: a systematic review and meta-analysis of prevalence

**DOI:** 10.1186/s11689-021-09362-5

**Published:** 2021-07-23

**Authors:** M. Marlborough, A. Welham, C. Jones, S. Reckless, J. Moss

**Affiliations:** School of Psychology, George Davis Centre, University of Leicester, Leicester, UK

**Keywords:** Fragile X syndrome, Female, Autistic spectrum disorder, Prevalence, Meta-analysis

## Abstract

**Background:**

Whilst up to 60% of males with fragile X syndrome (FXS) meet criteria for autism spectrum disorder (ASD), the prevalence and nature of ASD in females with FXS remains unclear.

**Method:**

A systematic literature search identified papers reporting ASD prevalence and/or symptomatology in females with FXS.

**Results and conclusion:**

Meta-analysis suggested that rates of ASD for females with FXS are reliably higher than for females in the general population (a random effects model estimated weighted average prevalence at 14%, 95% CI 13–18%). Whilst papers highlighted a number of social and repetitive difficulties for females with FXS, characteristic profiles of impairment are not clear. Possible associations between ASD traits and IQ, and between ASD and levels of fragile X mental retardation protein, are suggested, but data are equivocal.

## Background

Fragile X syndrome (FXS) is the most common inherited single-gene cause of intellectual disability and autism spectrum disorder (ASD). It is caused by a mutation on the X chromosome in the *FMR1* gene, typically due to the expansion of the CGG triplet repeat, resulting in disruption to the fragile X mental retardation protein (FMRP). FXS occurs when a person has 200 or more repeats (the full mutation); a repeat size between 55 and 200 is classified as a premutation. Cognition and behaviour are more severely affected for those with a full mutation, whose prevalence is approximately 1.4 per 10,000 males and 0.9 per 10,000 females according to a recent meta-analysis [1]. Due to the X-linked nature of the condition, females are reported to be less affected than males [2]. The increased severity of presentation in males and the lower prevalence in females has led to underrepresentation and often exclusion of females in research.

A prominent set of behavioural/psychological features associated with FXS are characteristics associated with ASD. The prevalence of ASD in the general population is approximately 1 in 68 [3], with boys being more commonly diagnosed (1 in 42) than girls (1 in 189). Rates of ASD in those with certain genetic neurodevelopmental syndromes are considerably higher, with the nature of ASD-related behaviours also reported to vary by syndrome group (e.g. [4–6]).

More than 90% of males with FXS are reported to display autistic-like characteristics and, when using gold standard diagnostic instruments, up to 60% of males meet diagnostic criteria [7–9]. Very few researchers have included females in their samples [10]. One review [4] found the prevalence of ASD for females with FXS was 1–3%; however, this was based on only two papers with female participants. Several studies have since been undertaken including female participants, necessitating further review.

For FXS, results across several studies suggest that children with comorbid ASD differ in their symptoms from children with FXS only, with the former being more similar to individuals with idiopathic autism than to individuals with FXS only [11–14]. However, given the low number of female participants, it is unclear whether these findings are also observed in females with FXS and ASD [10].

Two factors potentially associated with ASD in FXS are the level of intellectual disability and FMRP levels. There have been several studies which have reported that children diagnosed with FXS and ASD have significantly lower IQ scores than individuals with FXS without autism (e.g. [11, 14–17]). Less is known about any possible connection between IQ and ASD in females with FXS. Studies assessing the potential link between individuals’ level of FMRP and ASD-related behaviours have reported mixed results. Hessl et al. [18] found that FMRP did not predict autistic behaviours in those with FXS, but Hatton et al. [19] reported that lower levels of FMRP did predict higher scores for autistic behaviours. Since levels of FMRP and IQ are also correlated with each other [20, 21], the potential relationships between ASD, IQ and FRMP may be complex. However, again, the vast majority of research has been conducted with males.

The current paper presents a review of existing literature on the prevalence and nature of ASD in females with FXS. Prevalence data were meta-analysed to give a weighted average prevalence. The reported severity and nature of behaviours associated with ASD are also assessed, and we consider factors potentially associated with ASD behaviours or diagnosis, including IQ and FMRP levels.

## Method

### Initial search

Following PRISMA-P guidelines for systematic reviews [22], a systematic literature search was conducted in July 2018 of three online databases: PsychINFO (1984 to July 2018), PubMed (1948 to July 2018) and SCOPUS (1966 to July 2018). This search was repeated in February 2020 for the time period July 2018 to February 2020, as an update. Search terms (see Table 1) were determined from an initial scoping of literature and journals and followed up by investigating MeSH (Medical Subject Heading) term browsers and researching key terms used by The Fragile X Society and The National Autistic Society.

**Table 1:** Search terms

Fragile X Fragile X Syndrome Fragile-X FXS FRAXA Syndrome AFRAX Martin Bell* Syndrome Marker X Syndrome fraX Syndrome fra(X) Syndrome X-linked mental retardation Macroorchidism Escalante* Syndrome Escalante* FRAXE Syndrome Fragile X mental retardation Fragile X-F mental retardation Mar(X) Syndrome Mental retardation, X-linked *FMR1* FMRP	autis* autism* autistic* ASD autism spectrum disorder* PDD-NOS PDDNOS PDD unspecified PDD pervasive developmental disorder* pervasive developmental disorder not otherwise specified Asperger* Asperger* syndrome

Terms within each list were combined with OR operators; the two lists were combined with the AND operator

Where databases allowed, MeSH terms were also included. To ensure comprehensiveness of the search, no terms were included to denote sex, with papers including only male subjects excluded at a later stage. Initial filters were used to include only peer-reviewed journals available in the English language and to exclude studies with non-human subjects.

### Inclusion/exclusion criteria

Papers were selected for review if they reported behaviours related to ASD or prevalence of ASD in females with FXS. Papers were excluded if they (1) reported on FXS males only, (2) contained no analysis by sex, (3) focused solely on neurology, genetics, biology, a drug trial or development of a research measure, (4) did not report ASD behaviours or prevalence or (5) reported on FXS *within* samples of individuals with ASD.

Where it was not clear from the title/abstract whether a paper might meet the inclusion criteria, the full paper was read.

### Abstract and full paper review

**Fig. 1:**
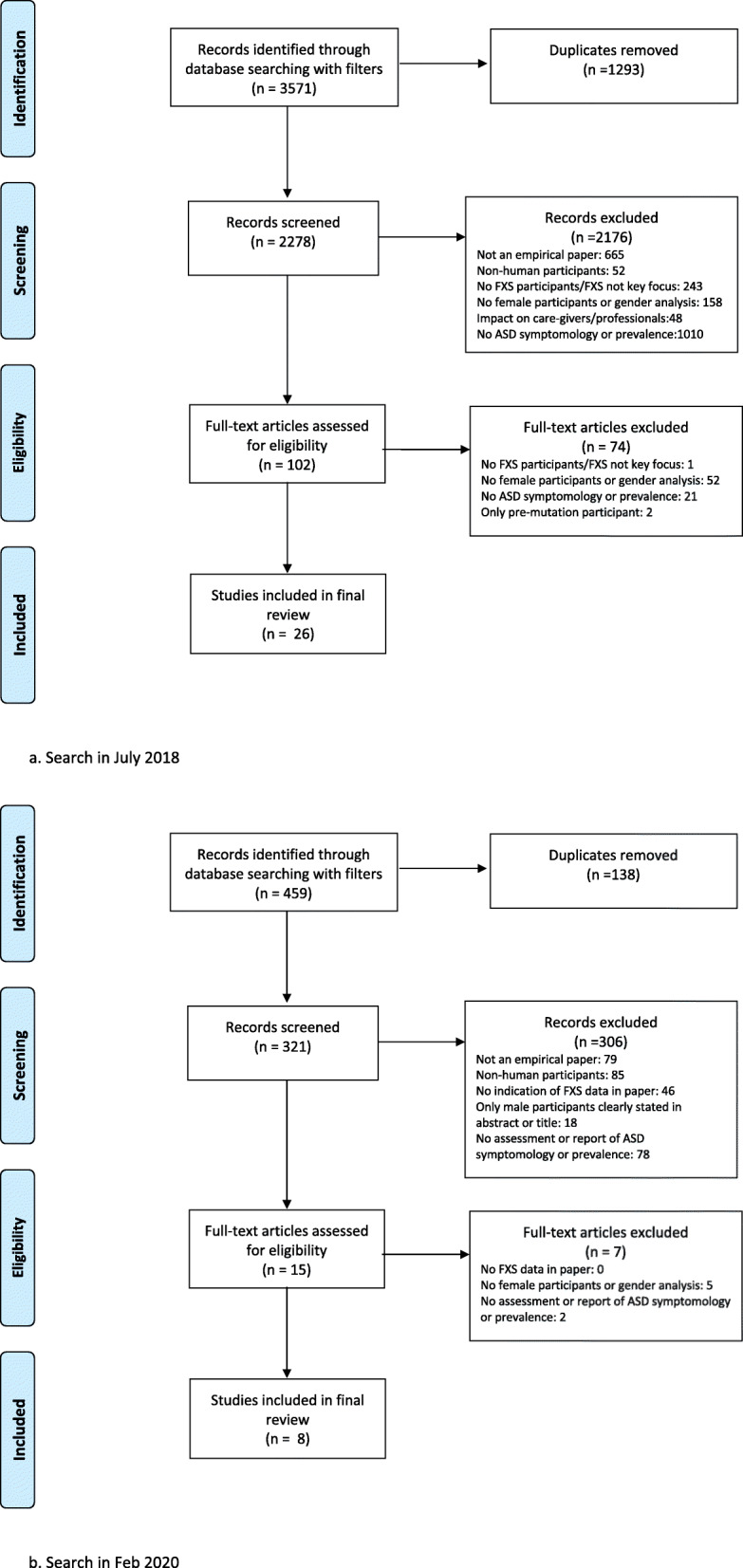
Flow diagrams of review process based on PRISMA group flow chart [22]. **a** Search in July 2018. **b** Search in Feb 2020

See Fig. 1 for PRISMA flowcharts summarising results of the search processes at the two timepoints. A total of 34 papers (28 up to July 2018 and 8 from July 2018 up to February 2020) were identified for inclusion.

Hand searching was also used to check references of the final papers, and a search was completed on Google to search the ‘grey literature’, which are materials and research produced by organisations outside of the traditional academic publishing channels. This may have included dissertations, non-published work, data provided by charities or other organisations. No additional papers were identified.

### Risk-of-bias/quality assessment

A risk-of-bias/quality rating tool was utilised that specifically appraises studies of ASD in genetic syndromes ([6]; see Table 2 for risk-of-bias criteria and visual colour coding). The tool addresses variability in risk of bias in ASD assessment (from parental report or screening tools to assessment using multiple ‘gold standard’ diagnostic measures), as well as potential sources of bias based on the nature of the sample or confirmation of diagnosis [6]. A score of 0–3 (with higher scores indicating higher quality/lower risk of bias) is given based on the study risk of bias in three areas: sample identification, confirmation of genetic syndrome and ASD assessment. A total score for each study, which could range from 0 to 9, was calculated as the sum of these three scores. The first author rated all papers for quality/risk-of-bias. Independent blind ratings were also obtained from a second rater (fourth author) for the calculation of inter-rater reliability. Agreement was excellent for individual domains, with weighted kappa calculated to be 0.80 (95% CI 0.53–1.1) for sample identification, 0.77 (95% CI 0.62–0.92) for ASD assessment and 0.75 (95% CI 0.58-0.93) for assessment of syndrome. For the total score, a two-way random effects, consistency, average-measures intraclass correlation [23, 24] also indicated high levels of agreement between raters (ICC .93, 95% CI .85–.96).

**Table 2:**
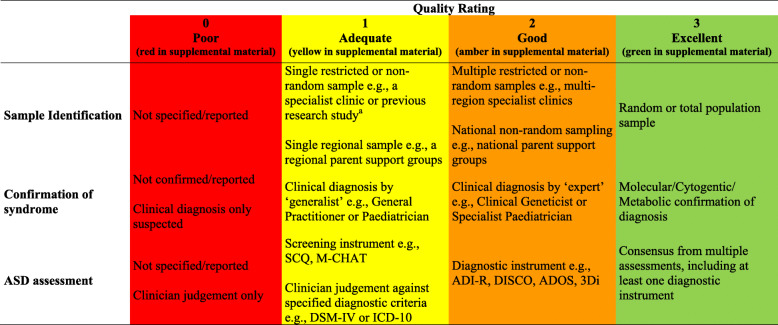
Quality/risk of bias criteria for sample identification, confirmation of syndrome and ASD assessment

^a^Where individuals were recruited as part of a larger ongoing study, if the recruitment strategy of this study is described, this is coded. If not described, it is coded as 1 by default, indicating the sample has come from one source (i.e. the larger ongoing study)

An overall quality/risk-of-bias weighting between 0 and 1 was calculated by dividing the total score by the maximum possible total score of nine. This score was used in the weighting of individual studies in the quality effects weighted average prevalence estimate and was considered when discussing the further findings. Ratings from the first author are the ratings presented in the paper and used in meta-analyses. However, quality effects meta-analyses were repeated, replacing ratings from the first author with ratings from the second author; these analyses confirmed that results did not change appreciably.

Overall quality/risk-of-bias assessment scores ranged from 0.11 to 0.78 (with higher scores indicating higher quality/lower risk of bias). In accordance with findings from Dixon-Woods et al. [25] and Stroup et al. [26], articles were not omitted due to risk-of-bias/quality (though see below in relation to meta-analysis).

### Data analysis

#### ASD prevalence

To determine the prevalence of ASD in females with FXS, the total number of females reported in the sample, and the number meeting cut-off for ASD were extracted from each paper. Where an assessment provided different cut-offs (e.g. Autistic Disorder rather than the broader spectrum cut-off), data regarding the most stringent cut-off level were used (following [6]). Similarly, where multiple assessments were used, data were extracted from the most robust assessment measure as determined by the risk-of-bias assessment tool used [6].

Meta-analytic weighted prevalence values were generated using a random effects model, selected to allow for between-study variation reflecting both sampling errors and other factors (such as variation in risk of bias in different methodologies) [27]. Since initial Q-Q plots indicated possible non-normality of distribution of prevalence estimates, the restricted maximum likelihood estimator was used to calculate between studies variance, due to its relative robustness to non-normal distributions of effects. A leave-one-out methodology, whereby each paper was omitted in turn and the weighted prevalence re-calculated, was used to identify studies of disproportionate influence (with the visual aid of Baujat charts). An additional quality effects model was also utilised, with adjusted weightings according to studies’ overall risk-of-bias ratings.

The existence of possible publication bias was assessed using the visual aid of a funnel plot, in which the magnitude of the study’s proportion estimate is plotted against the square root of the study’s sampling variances. If there is an absence of publication bias, the effects from the studies with small sample sizes which show greater variability will scatter more widely at the bottom of the plot compared to studies with larger samples at the top which will lie closer to the overall meta-analytic effect, creating a symmetrical funnel shape. If there is an absence of studies in the area of the plot associated with small sample sizes and low prevalence (for this meta-analysis it will be the bottom left-hand corner) then it is likely there is some publication bias leading to an overestimation of the true effect. Following Terrin et al. [28] demonstration of the unreliability of subjective judgments of funnel plot symmetry, Egger et al.’s [29] linear regression test of funnel plot asymmetry was also carried out. A trim and fill method was then used to model and correct for asymmetry due to potential publication bias [30, 31], producing adjusted weighted average prevalence estimates. The trim and fill procedure builds on the assumption that publication bias would lead to an asymmetrical funnel plot. It iteratively removes the most extreme small studies from the side of the funnel plot associated with positive effects, re-computing the effect size at each iteration until the funnel plot is symmetric about the (corrected) effect size. Whilst this trimming yields the adjusted effect size, it also reduces the variance of the effects, resulting in a biased and narrow confidence interval. Therefore, the original studies are returned into the analysis, and the procedure imputes a mirror image for each on the side of the funnel plot associated with small effect sizes. The *L*_0_ estimator was used in the current analysis, as outlined by Duval [32] and as is the default in the metafor (V2.4) procedure within R [33].

#### Nature of ASD: relationship of ASD with IQ and FMRP levels

Due to limitations in available statistical information, formal statistical analysis was not carried out for the review of the nature of ASD-related behaviours nor any associations with IQ or FMRP levels; therefore, synthesis is narrative in these areas.

## Results

There were 34 papers with findings pertaining to females with FXS (see Table 3 for a summary).

**Table 3:**
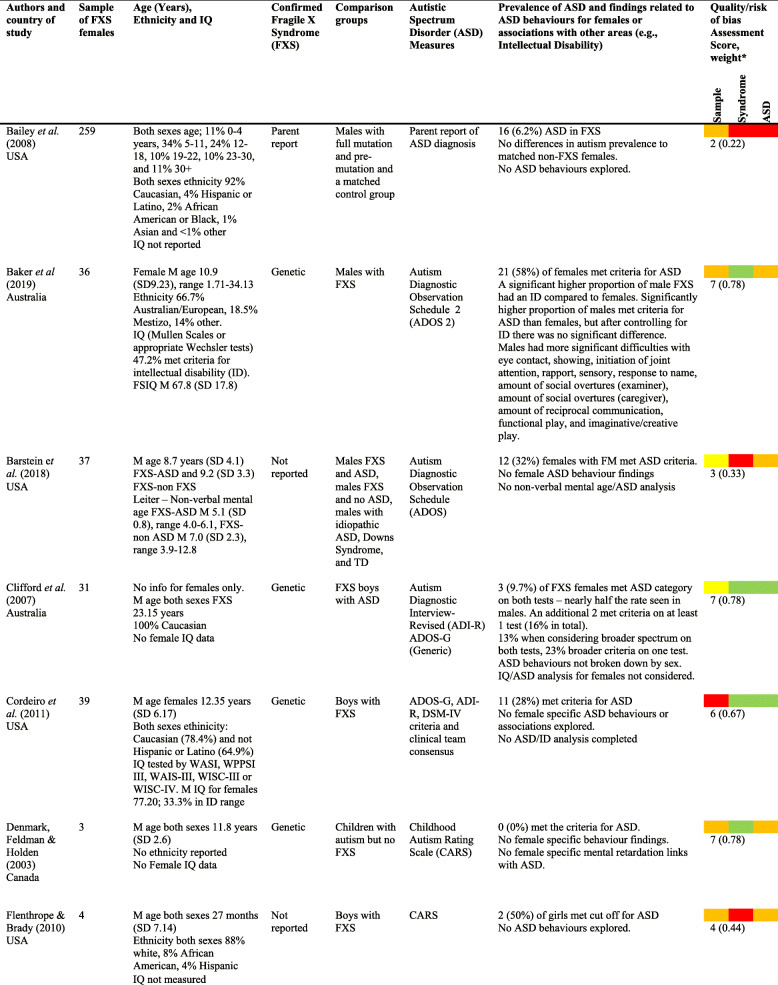
Main findings and papers from the review [10, 12, 18, 19, 34–64]

### Prevalence of ASD in females with FXS

**Fig. 2:**
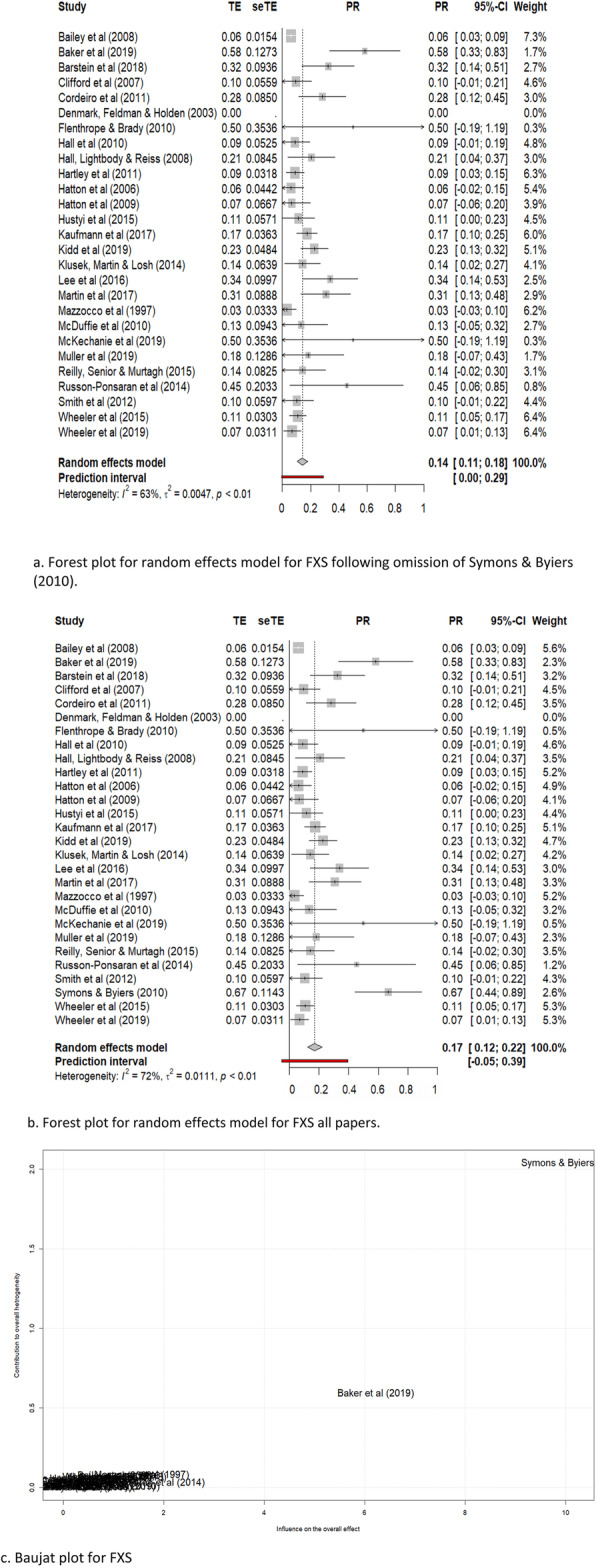
Forest plots of random effects models and Baujat plot of ASD in fragile X females. **a** Forest plot for random effects model for FXS following omission of Symons et al. [62]. **b** Forest plot for random effects model for FXS all papers. **c** Baujatplot for FXS

Twenty-eight papers reported prevalence data for ASD in females with FXS. Reported prevalence ranged from 0 to 66%. Weighted average prevalence of ASD among female participants with FXS was 17% (95% CI 12 to 22%; *z* = 6.8, *p* <.0001) for the random effects model, and the analysis indicated moderate heterogeneity (Higgins’ *I*^2^ = 72%; *τ*^2^ = .01; Q(26) = 92.0, *p* < .0001) (Fig. 2a). A leave-one-out analysis indicated that two papers—Symons et al. [62] and, to a lesser extent, Baker et al. [35]—may have exerted disproportionate influence in the analysis (see Baujat plot in Fig. 2c). Omitting Symons et al. [62] led to the greatest change in the meta-analytic estimate (15.8%). Symons et al. [62] also received ratings indicative of high risk of bias (i.e. low ratings on the quality assessment tool), especially with respect to confirmation of ASD. This was not the case for Baker et al. [35], whose risk-of-bias ratings indicated a high-quality study. A further analysis was therefore conducted without the inclusion of Symons et al. [62]. The revised weighted average prevalence estimate was 14% (95% CI 13 to 18%, *z* = 7.6, *p* < .0001), with reduced heterogeneity (*I*^2^ = 63%; *τ*^2^ = .005; *Q*(25) = 67.8, *p* < .0001) (see Fig. 2b).

For the quality effects model, weighted average prevalence was 22% (95% CI 16 to 29%; *z* = 6.9, *p* < .001; *I*^2^ = 82.4%; *τ*^2^ = .01 ; *Q*(26) = 92.0, *p* < .0001) using all papers, and 22% (95% CI 16 to 27%; *z* = 7.7, *p* <.0001; *I*^2^ = 67.2%; *τ*^2^ = .005; *Q*(25) = 67.8, *p* < .0001) after omission of the Symons et al. [62] paper.

**Fig. 3:**
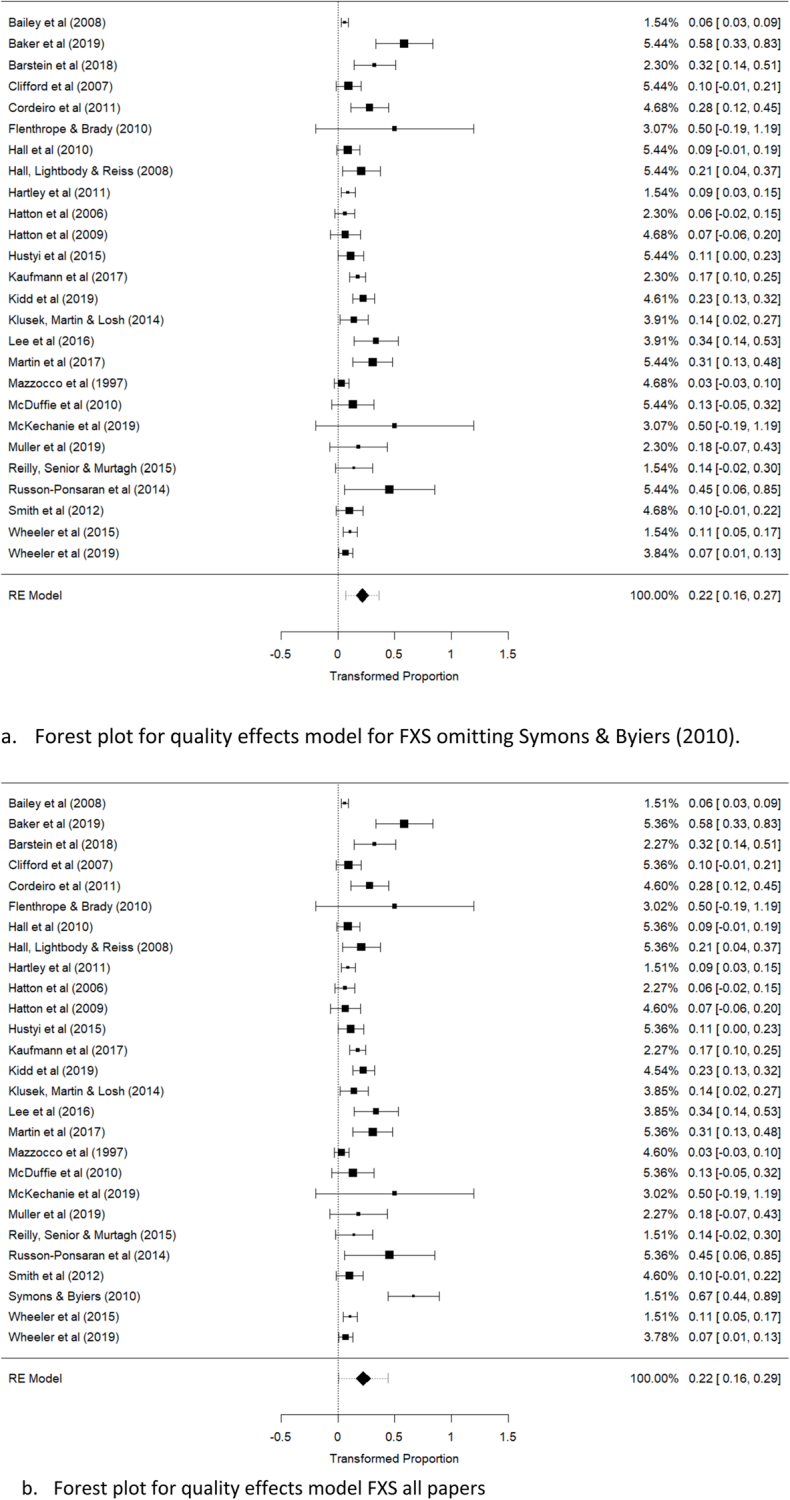
Forest plots of quality effects model of ASD in fragile X females. **a** Forest plot for quality effects model for FXS omitting Symons et al. [62]. **b** Forest plot for quality effects model FXS all papers

Forest plots for random and quality effects models, with and without the Symons et al. [62] paper, are shown in Figs. 2 and 3. A Baujat plot is also shown (Fig. 2c), illustrating the disproportionate influence of this specific paper.

**Fig. 4:**
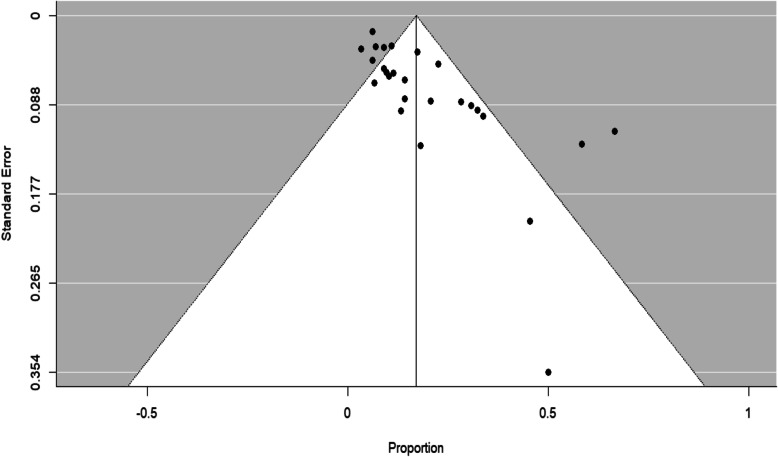
Funnel plot, in which studies' reported proportion of participants meeting criteria for ASD is plotted against the square root of the studies' sampling variance

A funnel plot (Fig. 4) indicated possible publication bias, a conclusion backed by Egger et al.’s [29] linear regression test of funnel plot asymmetry (bias 2.3, *t*(25) = 5.3, *p* < .0001). Using the trim and fill procedure [30, 31], eight studies were introduced, leading to an imputed estimate of the prevalence of 12% (95% CI 5–19%).

### Profile of behavioural characteristics associated with ASD

A number of papers reported on the frequency and topography of specific behaviours associated with ASD (see Table 4 for main findings from the papers exploring ASD-associated behaviours).

**Table 4:**
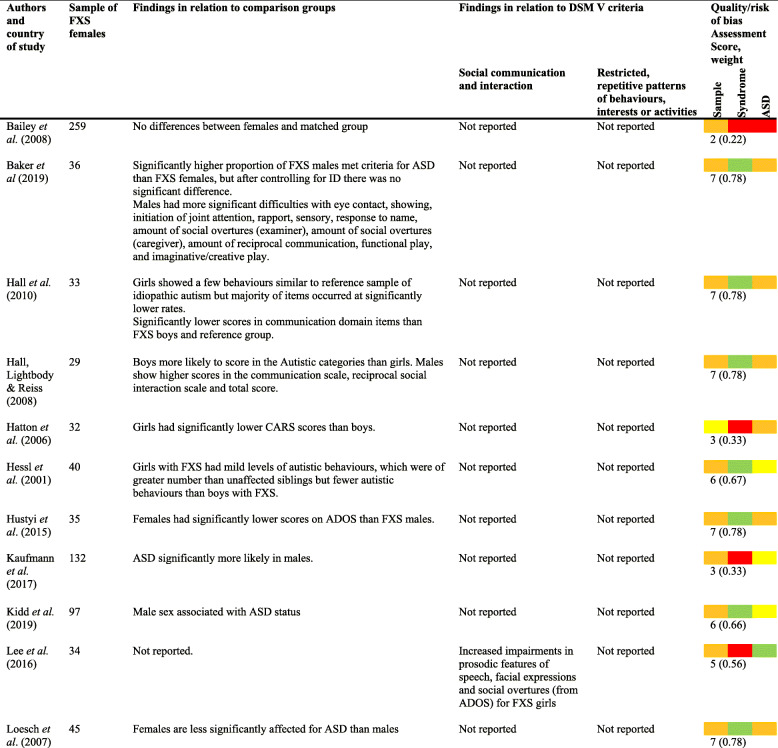
ASD behaviour findings [18, 19, 34, 35, 40, 41, 43–45, 47, 48, 50–52, 56, 59–63]

Studies generally reported that females with FXS showed fewer behaviours associated with ASD than the reported contrast groups, including males with FXS (e.g. [19, 35, 40, 43–45, 47, 56, 61, 62]). Interestingly, Baker et al. [35] found, in a paper rated high for quality, that whilst a greater proportion of males than females met criteria for ASD, this effect disappeared when controlling for the level of ID. Results also suggest that females with FXS were more likely to have ‘mild’ autistic behaviours, showing more characteristics than their unaffected siblings, unaffected typically developing peers and peers with other neurogenetic disorders but fewer characteristics than males with FXS [52, 56].

Females with FXS who met criteria for ASD were found to show similar rates and types of ASD symptomatology to individuals with idiopathic autism [40, 60] and to those described for males with FXS [52]. These appear to be robust findings from papers with relatively high ratings for quality (low risk of bias).

### Associations between IQ/cognitive ability and ASD characteristics

The majority of papers (17; 61%) did not report the cognitive ability of female participants, and correlations between IQ and ASD characteristics were also not reported. Eleven papers reported IQ levels or non-verbal mental age, but only six considered these in relation to ASD characteristics or diagnosis (see Table 5 for results and papers for IQ).

**Table 5:**
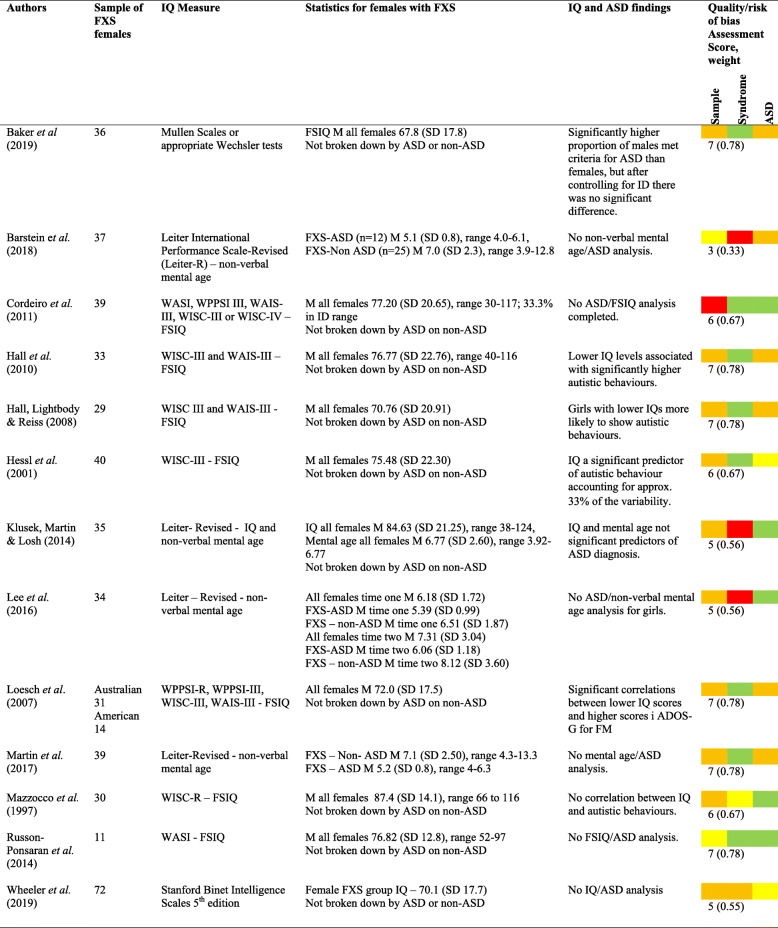
Results and papers for IQ and ASD [18, 35, 36, 38, 40, 41, 46–49, 52, 58, 64]

*TD* typically developing, *M* mean, *SD* standard deviation

*Colour code—red = poor, yellow = adequate, orange = good, green = excellent

Several studies scoring high on quality ratings reported a statistically significant negative association between IQ and autistic features in females with FXS, such that lower IQ scores were associated with significantly more autistic behaviours [18, 40, 41, 48]. In contrast, two studies reported that IQ and mental age were not significant predictors of ASD diagnosis or ASD characteristics in females with FXS [46, 52].

### Associations between FMRP levels and ASD characteristics

Six papers reported and analysed FMRP levels in relation to ASD characteristics or diagnosis (see Table 6). This is reported in all papers as the percentage of lymphocytes expressing FMRP.

**Table 6:**
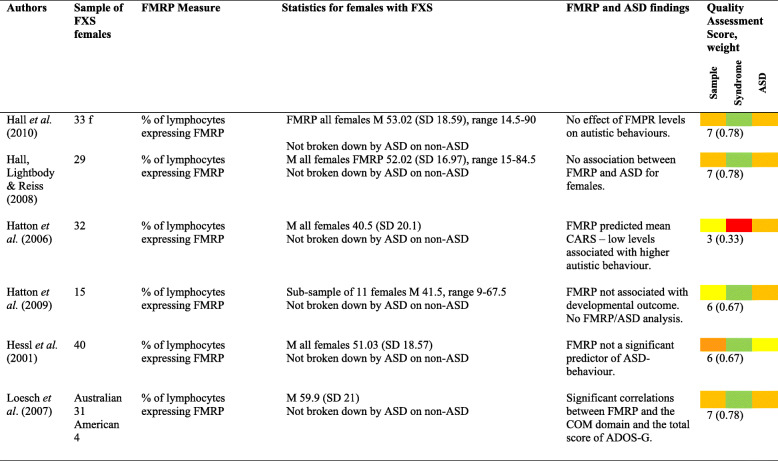
Results for FMRP and ASD [10, 18, 19, 40, 41, 48]

*Colour code—red = poor, yellow = adequate, orange = good, green = excellent

Mean FMRP scores ranged from 40.5 to 59.9%. Two papers suggested links between FMRP and autistic features [19, 48], whilst three papers reported no significant relationship [18, 40, 41].

### Additional variables potentially related to ASD

ASD in females with FXS was associated with greater levels of dependency and poorer developmental outcomes [10, 42, 43]. Anxiety and the presence of self-injurious behaviour was strongly correlated with ASD characteristics [52].

## Conclusions

This review examines and meta-analyses the prevalence of ASD among females with FXS. It also addresses the severity and nature of ASD characteristics in these groups, and evidence related to factors potentially associated with ASD, including IQ and FMRP levels. Data were reviewed from 34 studies. The quality/risk-of-bias of these studies was assessed using a published quality/risk-of-bias appraisal tool and considered as part of prevalence meta-analysis and in narrative interpretation of further findings.

### ASD in females with FXS

Published prevalence values were highly heterogenous, impeding confident interpretation of a single weighted average prevalence value based on all available papers (17%, 95% CI 12-22%). Following exclusion of a single disproportionately influential paper rated as having a high risk of bias [62], data were less heterogenous and a slightly lower weighted average prevalence (14%; 95% CI 13–18%) was estimated. When studies were additionally weighted by their quality/risk of bias, the estimate of prevalence was higher, at 22% (and was also relatively unchanged by omission/inclusion of the Symons and Byiers paper, reflecting in part its lower weight in the analysis due to its rating indicative of high risk of bias), although 95% confidence intervals (16 to 29%) overlap with those for the random effects model. Asymmetry of distribution of effects as observed in funnel plots may potentially reflect lack of reporting of ASD prevalence in smaller studies in which ASD prevalence was relatively low. If this is the case, then weighted average prevalence values in the uncorrected meta-analyses may represent over-estimates; models correcting for this possible bias produced slightly lower estimates (12%, 95% CI 5–19%). Overall, existing data do not allow a precise statement of a single meta-analytic prevalence value; however, an estimate taking into account studies’ risk of bias (the importance of which is highlighted by, e.g. [65]) suggests that over a fifth of females with FXS may meet criteria for ASD. Whether this represents an overestimate due to possible reporting bias should remain a focus of future research, in which care should be taken to publish ASD prevalence whether or not this is high within any particular study. It should be noted that the lower 95% confidence interval of every meta-analytic estimate of ASD prevalence for females with FXS was higher than reports in the general population (approximately 1 in 189 girls [3], or .53%), indicating that females with FXS are at increased risk for ASD. Lower 95% CIs also all exceeded the 1–3% estimate for females with FXS stated in a previous review [4]. These findings suggest that the FXS mutation increases ASD risk for females, perhaps to a greater degree than previously assumed, despite the potentially protective effect of the additional, unaffected X chromosome (whose influence may be demonstrated by the consistently lower levels of autistic behaviours found for girls than boys with FXS).

It is important to note that the instruments utilised for ASD diagnosis varied across studies, ranging from the parental report of diagnosis and broad screening measures to the ‘gold standard’ use of multiple comprehensive diagnostic instruments. It is well established that agreement between instruments can be variable. The studies outlined in this review indicate that this is also evident. For instance, Klusek et al. [46] reported significant differences in prevalence rates derived from the ADI-R and the ADOS, with 14.3% meeting the criteria on both the ADI-R and ADOS, 22.9% on the ADI-R but not the ADOS, and 25.7% for the ADOS but not the ADI-R.

ASD characteristics were reported in 21 papers, and included both social communication difficulties (e.g. difficulties both with non-verbal communication and language form, [47, 52, 63]) and repetitive/restrictive behaviours [52, 60, 63]. Differences in ASD characteristics relative to males with FXS [35, 36, 49, 58] were consistent with lower levels of atypicality (which in turn may relate to higher adaptive functioning) in girls. No papers reported greater levels of difficulty for girls than boys in any specific area. However, it remains possible that there are specific ASD-related clinical concerns for this group. Anxiety and the presence of self-injurious behaviour were both strongly correlated with ASD in this population [52]. These associations are also seen for people with idiopathic ASD and males with FXS [38, 66].

The findings were mixed when considering the associations between ASD and IQ and FMRP, with some studies indicating strong associations between ASD severity and IQ and FMRP levels and others reporting no correlations. Given that the nature of IQ assessment and sample sizes were similar across these studies, it is not clear why the resultant findings regarding IQ were inconsistent. Due to the focus on ASD in this paper, we reviewed potential relationships of FMRP and IQ with ASD symptomatology. However, it should be noted that IQ and FRMP are also associated with each other [20, 21], and levels of FMRP may be considered to underlie both ASD and low IQ [67] in FXS in general. Understanding of the possible interrelationships between the three variables for females with FXS is still relatively rudimentary, and it remains possible that knowledge of the ways in which FMRP, ASD and IQ interrelate in males with FXS does not entirely generalise to females. For example, if relationships between IQ and ASD are non-linear (as may be the case for idiopathic ASD, [68, 69]), then the different ability levels seen in girls and boys with FXS may mean that the relationship between IQ and ASD is also different for these two groups. Future research may continue to assess the strengths of linear associations (as has been generally undertaken) between FMRP, IQ and ASD in larger groups, and also may consider potentially non-linear aspects of these relationships.

### Strengths and limitations

The findings should be considered in light of several methodological constraints. Given the range of ASD diagnostic assessments used across studies, and the reported variability in sensitivity and specificity of these measurement tools, the prevalence data reported in this review should be considered as estimates only. Whilst the weighting of individual studies in the generation of quality weighted meta-analytic prevalence estimates is based partly on the risk of bias of the ASD measures, this cannot completely account for the wide and complex variability in ASD measurement in the reviewed papers. Further limitations relate to the use of the most stringent level of ASD assessment in each paper in the meta-analysis (a decision following Richards et al. [6], which allows for consistency with previous reviews, and replicability). Greater consistency within the literature in the stringency of reported ASD diagnosis may be important in the future.

Recruitment bias (e.g. for papers recruiting via specialist medical centres, participants may be more likely to be those with difficulties of clinical relevance) may also confound interpretation of prevalence estimates. Given the relatively small population of females with FXS, it is also possible that the same participants are included in more than one study, introducing further biases.

A large proportion of studies did not include appropriate contrast groups, as a large proportion only had males with FXS and the discrepancies between males and females with FXS are well-documented. Also, most contrast groups reported do not appear to be matched on IQ or age, which would also be important factors when considered appropriateness of controls. Future studies which are matched for gender, age and IQ would be most appropriate in order to not limit the findings.

The results reported are found for females across a wide variety of ages, with a few papers looking at ages across the lifespan but most having a focus on either children or adults. Research has shown differences in behaviours caused by FXS across the lifespan [70, 71], but none of the papers reviewed explored the impact of age ranges as potentially confounding factors, either in the analysis or discussion.

Strengths of this review include the systematic search strategy and use of a tool for risk-of-bias appraisal specifically developed for research into ASD in genetic syndromes, and with good levels of inter-rater reliability. Greater research focus on females with FXS is important in order to improve understanding and awareness of the challenges faced by affected individuals and their families.

## Data Availability

The data that support the findings of this study are available from the corresponding author upon reasonable request.

## References

[1] Hunter J, Rivero-Arias O, Angelov A, Kim E, Fotheringham I, Leal J (2014). Epidemiology of fragile X syndrome: a systematic review and meta-analysis. Am J Med Genet A.

[2] Lozano R, Hare EB, Hagerman RJ, Rosenberg RN, Pascual JM (2015). Fragile X-associated disorders. Rosenberg’s molecular and genetic basis of neurological and psychiatric disease.

[3] Centers for Disease Control and Prevention (2014). Prevalence of autism spectrum disorder among children aged 8 years. MMWR Surveill Summ.

[4] Moss J, Howlin P (2009). Autism spectrum disorders in genetic syndromes: implications for diagnosis, intervention and understanding the wider autism spectrum disorder population. J Intellect Disabil Res.

[5] Moss J, Howlin P, Oliver C, Burack J, Hodapp R, Iarocci G, Zigler E (2011). The assessment and presentation of autism spectrum disorder and associated characteristics in individuals with severe intellectual disability and genetic syndrome. The Oxford handbook of intellectual disability and development.

[6] Richards C, Jones C, Moss J, Groves L, Oliver C (2015). The prevalence of autism spectrum disorder phenomenology in genetic disorders: a systematic review and meta-analysis. Lancet Psychiatry.

[7] Harris SW, Hessl D, Goodlin-Jones B (2008). Autism profiles of males with fragile X syndrome. Am J Ment Retard.

[8] McDuffie A, Thurman AJ, Hagerman RJ, Abbeduto L (2015). Symptoms of autism in males with fragile X syndrome: a comparison to nonsyndromic ASD using current ADI-R scores. J Autism Dev Disord.

[9] Talisa VB, Boyle L, Crafa D, Kaufmann WE (2014). Autism and anxiety in males with fragile X syndrome: an exploratory analysis of neurobehavioral profiles from a parent survey. Am J Med Genet A.

[10] Hatton DD, Wheeler A, Sideris J, Sullivan K, Reichardt A, Roberts J (2009). Developmental trajectories of young girls with fragile X syndrome. Am J Intellect Dev Disabil.

[11] Bailey DB, Mesibov GB, Hatton DD, Clark RD, Roberts JE, Mahew L (1998). Autistic behavior in young boys with fragile X syndrome. J Autism Dev Disord.

[12] Demark J, Feldman M, Holden J (2003). Behavioral relationship between autism and fragile X syndrome. Am J Ment Retard.

[13] Lewis P, Abbeduto L, Murphy M, Giles N, Schroeder S, Bruno L (2006). Cognitive, language and social cognitive skills of individuals with fragile X syndrome with and without autism. J Intellect Disabil Res.

[14] Rogers S, Wehner E, Hagerman R (2001). The behavioral phenotype in fragile X: symptoms of autism in very young children with fragile X syndrome, idiopathic autism, and other developmental disorders. J Dev Behav Pediatr.

[15] Hagerman RJ, Jackson AW, Levitas A, Rimland B, Braden M (1986). An analysis of autism in fifty males with the fragile X syndrome. Am J Med Genet.

[16] Hernandez RN, Feinberg RL, Vaurio R, Passanante NM, Thompson RE, Kaufmann WE (2009). Autism spectrum disorder in fragile X syndrome: a longitudinal evaluation. Am J Med Genet A.

[17] Kaufmann WE, Cortell R, Kau AS (2004). Autism spectrum disorder in fragile X syndrome: communication, social interaction, and specific behaviors. Am J Med Genet A.

[18] Hessl D, Dyer-Friedman J, Glaser B, Wisbeck J, Barajas RG, Taylor A (2001). The influence of environmental and genetic factors on behavior problems and autistic symptoms in boys and girls with fragile X syndrome. Pediatrics..

[19] Hatton DD, Sideris J, Skinner M, Mankowski J, Bailey DB, Roberts J (2006). Autistic behavior in children with fragile X syndrome: prevalence, stability, and the impact of FMRP. Am J Med Genet A.

[20] Kovacs T, Kelemen O, Keri S (2013). Decreased fragile X mental retardation protein (FMRP) is associated with lower IQ and earlier illness onset in patients with schizophrenia. Psychiatry Res.

[21] Tassone F, Hagerman RJ, Ikle DN, Dyer PN, Lampe M, Willemsen R (1999). FMRP expression as a potential prognostic indicator in fragile X syndrome. Am J Med Genet.

[22] Moher D, Shamseer L, Clarke M, Ghersi D, Liberati A, Petticrew M (2015). Preferred Reporting Items for Systematic Review and Meta-Analysis Protocols (PRISMA-P) 2015 statement. Syst Rev.

[23] Hallgren KA (2012). Computing inter-rater reliability for observational data: an overview and tutorial. Tutor Quant Methods Psychol.

[24] McGraw K, Wong S (1996). Forming inferences about some intraclass correlation coefficients. Psychol Methods.

[25] Dixon-Woods M, Ashcroft RE, Jackson CJ, Tobin MD, Kivits J, Burton PR (2007). Beyond “misunderstanding”: written information and decisions about taking part in a genetic epidemiology study. Soc Sci Med.

[26] Stroup DF, Berlin JA, Morton SC, Olkin I, Williamson GD, Rennie D (2000). Meta-analysis of observational studies in epidemiology: a proposal for reporting. Jama..

[27] Hedges LV, Vevea JL (1998). Fixed-and random-effects models in meta-analysis. Psychol Methods.

[28] Terrin N, Schmid CH, Lau J (2005). In an empirical evaluation of the funnel plot, researchers could not visually identify publication bias. J Clin Epidemiol.

[29] Egger M, Smith GD, Schneider M, Minder C (1997). Bias in meta-analysis detected by a simple, graphical test. Br Med J.

[30] Duval S, Tweedie R (2000). Trim and fill: a simple funnel-plot-based method of testing and adjusting for publication bias in meta-analysis. Biometrics..

[31] Duval S, Tweedie R (2000). A nonparametric ‘trim and fill’ method of accounting for publication bias in meta-analysis. J Am Stat Soc.

[32] Duval S (2005). The trim and fill method. Publication bias in meta-analysis: prevention, assessment and adjustments.

[33] Viechtbauer W (2010). Conducting meta-analyses in R with the metafor package. J Stat Softw.

[34] Bailey DB, Raspa M, Olmsted M, Holiday DB (2008). Co-occurring conditions associated with *FMR1* gene variations: findings from a national parent survey. Am J Med Genet A.

[35] Baker EK, Arpone M, Vera SA, Bretherton L, Ure A, Kraan CM (2019). Intellectual functioning and behavioural features associated with mosaicism in fragile X syndrome. J Neurodev Disord.

[36] Barstein J, Martin GE, Lee M, Losh M (2018). A duck wearing boots?! Pragmatic language strategies for repairing communication breakdowns across genetically based neurodevelopmental disabilities. J Speech Language Hearing Res.

[37] Clifford S, Dissanayake C, Bui QM, Huggins R, Taylor AK, Loesch DZ (2007). Autism spectrum phenotype in males and females with fragile X full mutation and premutation. J Autism Dev Disord.

[38] Cordeiro L, Ballinger E, Hagerman R, Hessl D (2011). Clinical assessment of DSM-IV anxiety disorders in fragile X syndrome: prevalence and characterization. J Neurodev Disord.

[39] Flenthrope JL, Brady NC (2010). Relationships between early gestures and later language in children with fragile X syndrome. Am J Speech-Lang Pathol.

[40] Hall SS, Lightbody AA, Hirt M, Rezvani A, Reiss AL (2010). Autism in fragile X syndrome: a category mistake?. J Am Acad Child Adolesc Psychiatry.

[41] Hall SS, Lightbody AA, Reiss AL (2008). Compulsive, self-injurious, and autistic behavior in children and adolescents with fragile X syndrome. Am J Ment Retard.

[42] Hartley SL, Seltzer MM, Raspa M, Olmstead M, Bishop E, Bailey DB (2011). Exploring the adult life of men and women with fragile X syndrome: results from a national survey. Am J Intellect Dev Disabil.

[43] Hustyi KM, Hall SS, Quintin E, Chromik LC, Lightbody AA, Reiss AL (2015). The relationship between autistic symptomatology and independent living skills in adolescents and young adults with fragile X syndrome. J Autism Dev Disord.

[44] Kaufmann WE, Kidd SA, Andrews HF, Budimirovic DB, Esler A, Haas-Givler B (2017). Autism spectrum disorder in fragile X syndrome: cooccurring conditions and current treatment. Pediatrics..

[45] Kidd SA, Berry-Kravis E, Choo TH, Chen C, Esler A, Hoffmann A (2020). Improving the diagnosis of autism spectrum disorder in fragile X syndrome by adapting the social communication questionnaire and the social responsiveness scale-2. J Autism Dev Disord.

[46] Klusek J, Martin GE, Losh M (2014). Consistency between research and clinical diagnoses of autism among boys and girls with fragile X syndrome. J Intellect Disabil Res.

[47] Lee M, Martin GE, Berry-Kravis E, Losh M. A developmental, longitudinal investigation of autism phenotypic profiles in fragile X syndrome. J Neurodev Disord. 2016;8(1). 10.1186/s11689-016-9179-0 PMC5203725 28050218

[48] Loesch DZ, Bui QM, Dissanayake C, Clifford S, Gould E (2007). Bulhak-Paterson, et al. Molecular and cognitive predictors of the continuum of autistic behaviours in fragile X. Neurosci Biobehav Rev.

[49] Martin GE, Barstein J, Hornickel J, Matherly S, Durante G, Losh M (2017). Signaling of noncomprehension in communication breakdowns in fragile X syndrome, Down syndrome, and autism spectrum disorder. J Commun Disord.

[50] Martin GE, Barstein J, Patel S, Lee M, Henry L, Losh M (2019). Longitudinal analysis of communication repair skills across three neurodevelopmental disabilities. Int J Lang Commun Disord.

[51] Martin GE, Bush L, Klusek J, Patel S, Losh M (2018). A multimethod analysis of pragmatic skills in children and adolescents with fragile X syndrome, autism spectrum disorder, and Down syndrome. J Speech Language Hearing Res.

[52] Mazzocco MMM, Kates WR, Baumgardner TL, Freund LS, Reiss AL (1997). Autistic behaviors among girls with fragile X syndrome. J Autism Dev Disord.

[53] McDuffie A, Abbeduto L, Lewis P, Kim J-S, Kover ST, Weber A (2010). Autism spectrum disorder in children and adolescents with fragile X syndrome: within-syndrome differences and age-related changes. Am J Intellect Dev Disabil.

[54] McKechanie AG, Campbell S, Eley S, Stanfield AC (2019). Autism in fragile X syndrome; a functional MRI study of facial emotion-processing. Genes..

[55] Muller K, Brady NC, Warren SF, Fleming KK (2019). Mothers’ perspectives on challenging behaviours in their children with fragile X syndrome. J Intellect Develop Disabil.

[56] Reilly C, Senior J, Murtagh L (2015). ASD, ADHD, mental health conditions and psychopharmacology in neurogenetic syndromes: parent survey. J Intellect Disabil Res.

[57] Roid G, Miller L (1997). Leiter international test of intelligence—revised.

[58] Russo-Ponsaran N, Berry-Kravis E, McKown CA, Lipton M (2014). A pilot study of social information processing skills in girls with fragile X syndrome. J Ment Health Res Intellect Disabil.

[59] Shaffer RC, Schmitt L, John Thurman A, Abbeduto L, Hong M, Pedapati E (2020). The relationship between expressive language sampling and clinical measures in fragile X syndrome and typical development. Brain Sci.

[60] Smith LE, Barker ET, Seltzer MM, Abbeduto L, Greenberg JS (2012). Behavioral phenotype of fragile X syndrome in adolescence and adulthood. Am J Intellect Dev Disabil.

[61] Smith LE, Hong J, Greenberg JS, Mailick MR (2016). Change in the behavioral phenotype of adolescents and adults with FXS: role of the family environment. J Autism Dev Disord.

[62] Symons FJ, Byiers BJ, Raspa M, Bishop E, Bailey DB (2010). Self-injurious behavior and fragile X syndrome: findings from the national fragile X survey. Am J Intellect Dev Disabil.

[63] Wheeler AC, Mussey J, Villagomez A, Bishop E, Raspa M, Edwards A (2015). DSM-5 changes and the prevalence of parent-reported autism spectrum symptoms in fragile X syndrome. J Autism Dev Disord.

[64] Wheeler AC, Wylie A, Raspa M, Villagomez A, Miller K, Edwards A (2019). Decisional capacity for informed consent in males and females with fragile X syndrome. J Autism Dev Disord.

[65] Higgins JP, Thompson SG, Spiegelhalter DJ (2009). A re-evaluation of random-effects meta-analysis. J Royal Stat Soc Ser A.

[66] Wood J, Drahota A, Sze K, Har K, Chiu A, Langer D (2009). Cognitive behavioral therapy for anxiety in children with autism spectrum disorders: a randomized, controlled trial. J Child Psychol Psychiatry.

[67] Kover ST, Pierpont EI, Kim JS, Brown WT, Abbeduto LA (2013). A neurodevelopmental perspective on the acquisition of nonverbal cognitive skills in adolescents with fragile X syndrome. Dev Neuropsychol.

[68] Charman T, Pickles A, Simonoff E, Chandler S, Loucas T, Baird G (2011). IQ in children with autism spectrum disorders: data from the Special Needs and Autism Project (SNAP). Psychol Med.

[69] Wang HZ, Qin HD, Guo W, Samuels J, Shugart YY (2013). New insights into the genetic mechanism of IQ in autism spectrum disorders. Front Genet.

[70] Bailey JDB, Hatton DD, Tassone F, Skinner M, Taylor AK (2001). Variability in FMRP and early development in males with fragile X syndrome. Am J Ment Retard.

[71] Roberts JE, Mirrett P, Burchinal M (2001). Receptive and expressive communication development of young males with fragile X syndrome. Am J Ment Retard.

